# A comparative study of natural immune responses against Plasmodium vivax C-terminal merozoite surface protein-1 (PvMSP-1) and apical membrane antigen-1 (PvAMA-1) in two endemic settings

**DOI:** 10.17179/excli2015-388

**Published:** 2015-08-06

**Authors:** Hui Xia, Qiang Fang, Kulachart Jangpatarapongsa, Tao Zhiyong, Liwang Cui, Baiqing Li, Rachanee Udomsangpetch

**Affiliations:** 1 Department of Parasitology, Bengbu Medical College, Anhui 233030, China; 2 Anhui Key Laboratory of Infection and Immunity at Bengbu Medical College, Anhui 233030, China; 3 Center for Research and Innovation, Faculty of Medical Technology, Mahidol University, Bangkok 10700, Thailand; 4 Department of Clinical Microbiology and Applied Technology, Faculty of Medical Technology, Mahidol University, Bangkok 10700, Thailand; 5 Department of Entomology, Pennsylvania State University, PA 16802, USA; 6 Department of Immunology, Bengbu Medical College, Anhui 233030, China

**Keywords:** malaria immunity, Plasmodium vivax, Plasmodium falciparum, antibody to malaria

## Abstract

The mechanisms of cellular and humoral immune responses against *P. vivax* parasite remain poorly understood. Several malaria immunological studies have been conducted in endemic regions where both *P. falciparum* and *P. vivax* parasites co-exist. In this study, a comparative analysis of immunity to *Plasmodium vivax* antigens in different geography and incidence of *Plasmodium spp*. infection was performed. We characterised antibodies against two *P. vivax* antigens, PvMSP-1 and PvAMA-1, and the cross-reactivity between these antigens using plasma from acute malaria infected patients living in the central region of China and in the western border of Thailand. *P. vivax* endemicity is found in central China whereas both *P. vivax* and *P. falciparum* are endemic in Thailand. There was an increased level of anti-PvMSP-1/anti-PvAMA-1 in both populations. An elevated level of antibodies to total *P. vivax* proteins and low level of antibodies to total *P. falciparum* proteins was found in acute *P. vivax* infected Chinese, suggesting antibody cross-reactivity between the two species. *P. vivax* infected Thai patients had both anti-*P. vivax* and anti-*P. falciparum* antibodies as expected since both species are present in Thailand. More information on humoral and cell mediated immunity during acute *P. vivax*-infection in the area where only single *P. vivax* species existed is of great interest in the relation of building up anti-disease severity caused by *P. falciparum*. This knowledge will support vaccine development in the future.

## Introduction

Malaria remains one of the most devastating infectious diseases due to its influence on more than 225 million individuals and approximately 700 thousand deaths each year. The majority of malaria infections are caused by *Plasmodium falciparum* in African countries (Sachs and Malaney, 2002[20]). However, as the most widely distributed human malaria parasite, *Plasmodium vivax* can also cause life-threatening symptoms even though it was previously considered benign. In contrast to *P. falciparum*, studies on *P. vivax* have lagged behind, largely due to the fact that this parasite cannot be cultured continuously *in vitro*. The mechanisms of cellular and humoral immune responses against *P. vivax* parasite also remain poorly understood. Other reasons for the neglect of “benign” *P. vivax* malaria could be the difficulty in accessing *P. vivax*-infected cases. Several studies have been conducted in endemic regions where both parasites co-exist (Snounou and White, 2004[23]; Rodrigues et al., 2005[18]; Oliveira et al., 2006[15]; Wickramarachchi et al., 2007[29]; Michon et al., 2007[13]; Chuangchaiya et al., 2010[3]; Kochar et al., 2010[9]; Lin et al., 2010[10]; Douglas et al., 2011[6]; Lin et al., 2011[11]). The strain-specific and serological cross-reactive immunity between *P. falciparum* and *P. vivax* blood stage antigens has been documented (Diggs and Sadun, 1965[4]; Woodberry et al., 2008[31]; Doolan et al., 2009[5]). However, very few studies were conducted in areas where only *P. vivax* causes infection (Jangpatarapongsa et al., 2012[8]). Therefore, a comparative study of immunity to *P. vivax* antigens in different endemic settings will contribute to a better understanding on the development and dynamics of host immunity to *P. vivax* infections. 

Strong humoral immune responses to *Plasmodium* can be induced in residents of malaria endemic areas (Wipasa et al., 2002[30]) The level of total antimalarial antibodies increases with age and depends on the length and intensity of exposure to malaria. Antibody-mediated inhibition of parasites is more efficient in blood stage than in liver stage infections (Troye-Blomberg and Perlmann, 1988[27]). Antibodies also mediate antibody-dependent cellular cytotoxicity and phagocytosis involving polymorphonuclear cells, neutrophils or platelets (Bolad and Berzins, 2000[2]). 

To understand the natural immune response during *P. vivax* infection in central China where only *P. vivax* is present and western Thailand with *P. vivax* and *P. falciparum* were almost equally prevalent (WHO, 2013[28]), we determined antibodies in the patients' sera against proteins extracted from *P. vivax* parasites and recombinant proteins PvMSP1(19) and PvAMA-1 produced in Escherichia coli (Soares et al., 1997[25]; 1999[24][26]; Rodrigues et al., 2003[17]). Our study aimed to characterize the level of IgG antibodies following *P. vivax* infection comparing two malaria endemic areas having different geography and incidence of *Plasmodium spp.* infection.

## Materials and Methods

### Study population

Plasma samples were collected from 76 patients with acute *P. vivax* infections (AC) at Wuhe County Hospital, Guzhen County Hospital, The First City Hospital, Bengbu city, Anhui Province, China. The patients were enrolled sequentially during June and July of 2009 and 2010. All patients enrolled in this study are inhabitants of Wuhe County, Guzhen County or the Bengbu City suburbs. Malaria transmission in this region is non-stable but can lead to malaria endemic in China. In the 1960s and 1970s, there were two malaria epidemics which were primarily caused by the *P. vivax* parasite. *P. falciparum* and *P. vivax* parasites were found together in this region until the end of the 1980s, but *P. falciparum* has not been found since the early 1990s. During the first decade of this century (from 2000 to 2010), malaria in this and other regions of China was mainly caused by the *P. vivax* parasite. 

In Thailand plasma samples were collected from 52 patients from malaria clinics at Mae Sot and Mae Kasa, Tak Province, who were enrolled sequentially during 2009 and 2010. The diagnosis of *P. vivax* malaria infection was based on the examination of Giemsa-stained thick and thin blood films. Polymerase chain reaction (PCR) with species-specific primers was performed on DNA isolated from the blood samples to further verify *P. vivax* infections (Snounou et al., 1993[22]). 

Blood samples were collected from 32 Chinese and 53 Thai people who did not suffer from *P. vivax* at the time of blood collection determined by both microscopy and PCR residing in the same *P. vivax*-endemic area as “immune controls” (IC). Another 20 healthy Chinese adults living in Wuxi city, China and 27 healthy Thai adults living in Bangkok, Thailand without previous malaria exposure were recruited to serve as “naïve controls” (NC). The clinical characteristics of the subjects are listed in Table 1(Tab. 1). This study was approved by Ethical Approval Committee of Biomedical Institute of Anhui Medical University and Committee on Human Rights Related to Human Experimentation, Mahidol University. Informed consents were obtained from each individual before a blood sample was taken.

### Parasite culture and antigen preparation

*P. vivax*-infected red blood cells (iRBC) purified from infected blood were used as crude antigens for coating. Briefly, *P. vivax* infected bloods were depleted of white blood cells by filtering through a sterile column of CF11 cellulose (Whatman^®^, Maidstone, UK) and the red blood cells were washed with RPMI-1640 by centrifugation at 1190 g for 5 minutes. The parasites were cultured for 24 - 30 hours at 5 % hematocrit in McCoy's 5A medium (GIBCO, Carlsbad, USA) supplemented with 25 % human AB serum. *P. vivax* parasites were maintained in an incubator containing 5 % CO_2_, 5 % O_2_ and 90 % N_2_ until matured to schizont stage ( 6 nuclei). *P. faciparum* culture was performed as described previously (Jangpatarapongsa et al., 2006[7]). The late stage iRBC were enriched by centrifugation using 60 % Percoll (GE Healthcare, Uppsala, Sweden) at 1190 g for 10 minutes. The enriched iRBC pellets were sonicated for 40 seconds at 150 watts and the protein concentration was determined by Bradford assay (Bio-Rad, Hercules, USA). The proteins were then aliquoted and stored at -70 °C until use. Uninfected RBCs were processed similarly as above and equivalent amount of protein concentration to the malaria antigens was stored at -70 °C to be used as a negative control.

### Protein expression and purification

A blood filter paper from *P. vivax*-infected patients from Thailand was used to extract genomic DNA. PCR was used to obtain the MSP-1(19) fragment by using primers in Table 2(Tab. 2). Sequences were cut with *BamH I* and *Xho I* and cloned into pET28a vector. Protein was expressed in *E. coli* BL21. IPTG was used for protein induction and cell pellet was collected and sonicated. Protein was purified by using native condition as described by manufacturer's protocol (Qiagen, USA). Supernatant was collected and protein was purified by Ni-NTA beads. Finally, eluted protein was checked for expression by SDS-PAGE and kept at -20 °C. 

For PvAMA-1, PCR was performed to amplify PvAMA-1 gene from a Thai *P. vivax* sample by using the designed primers in Table 2(Tab. 2). Sequences were cut using *Nde I* and *Xho I* and cloned into pET22b vector. Protein was expressed in *E. coli* BL21, purified and checked for expression in similar processes to PvMSP-1(19) as mentioned above. 

### ELISA analysis and IgG antibody level in plasma

There were three groups of plasma samples analyzed: AC group (n = 76), IC group (n = 32), and NC group (n = 20). There were five soluble antigens used: NRBC (10 μg/ ml), PV (10 μg/ml), PF (10 μg/ml), PvMSP-1 (0.25 μg/ml), and PvAMA-1 (0.1 μg/ml). 50 μl of antigens were pipetted to each ELISA well and plates were stored in wetted box at 4 °C overnight. Then the plates were washed and blocked with 5 % skimmed milk for 2 h at 37 °C. Plasma samples were diluted (1: 100) and added to each well (50 μl). The plates were enclosed in wetted box and incubated for 2 h at 37 °C. After washing 3 times using TBS-T, 50 μl of HRP-conjugated goat anti human IgG (H&L chain) antibody was added into each well, then plates were enclosed in wetted box again, and incubated for another 2 h at 37 °C. TBS-T was used to wash the plate then 50 μl of ABST substrate were added to each well. The plate was stored in the dark for 1 h at room temperature. All absorption was determined at A405 and the final OD values of PV and PF antigen were obtained by subtracting the OD values from NRBC group.

## Results

### A comparison of anti-P. vivax antibody after being intected with P. vivax parasite between Thai and Chinese patients

#### Anti-crude PV

**Figure 1:**
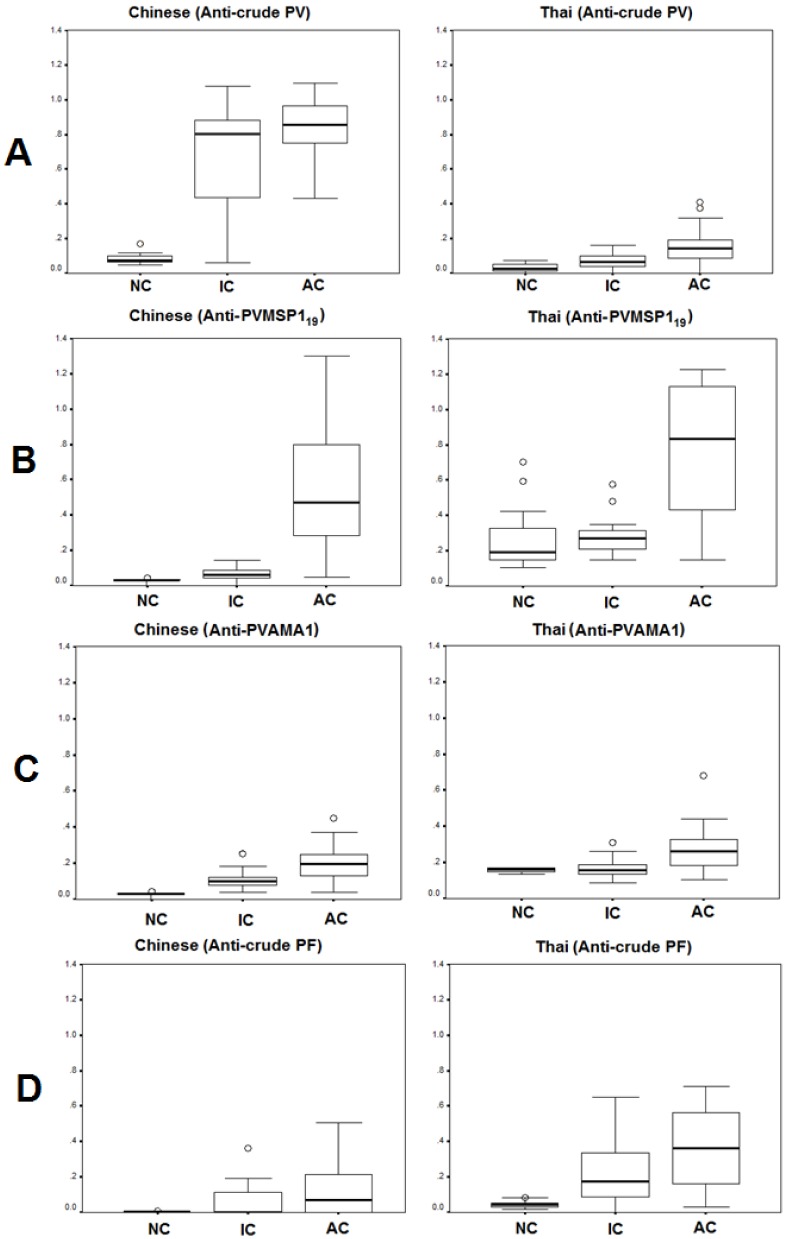
Absorbance (405 nm) value of IgG antibody reacting with (A) crude *P. vivax* antigens, (B) recombinant *P. vivax* MSP-1_19_ protein, (C) recombinant *P. vivax* AMA-1 protein, and (D) crude *P. falciparum* antigen respectively, in the naïve controls (NC), immune controls (IC), acute *P. vivax* infection (AC) comparing between Chinese and Thai patients. Data are shown in median, interquartile ranges (box plots), maximum and minimum (upper-lower lines).

There was significant difference of base line antibody level among naïve controls against *P. vivax*-extracted antigens between Chinese (OD = 0.07) and Thai (OD = 0.03) (MD = 0.07, 95 % CI = 0.04-0.1, P < 0.001) (Figure 1A(Fig. 1)). After infection by *P. vivax*, significant elevation of antibodies were found in both patient groups Chinese (0.85) and Thai (0.14). However, the level of antibody in *P. vivax* infected Chinese patients was higher than that of the Thai patients. Similar to naïve controls, the immune Chinese controls (0.8) had higher levels of antibodies against the *P. vivax* antigens than that of the Thai controls (0.07).

#### Anti-P. vivax MSP-1(19) 

Baseline values of mean level of antibodies to PvMSP-1(19) among healthy Thai controls and Chinese population living outside (naïve controls) and inside (immune controls) endemic areas were compared (Figure 1B(Fig. 1)). We found that the mean level of total antibodies in Thai naïve controls (OD = 0.3) was higher than that of Chinese naïve controls (OD = 0.03) (P < 0.001). Moreover, the level of antibodies in people living in *P. vivax* endemic area was significantly different between Thai (0.31) and Chinese populations (0.06) (P < 0.001). Two samples of Thai healthy controls had antibodies to PvMSP-1(19) as high as *P. vivax* infected patients as shown in above figure(Fig. 1), and were excluded from the baseline value. Among both population groups, the mean level of total antibodies to PvMSP-1(19) was significantly increased during acute *P. vivax* infection in both Thai (0.8) (P < 0.001) and Chinese (0.5) (P < 0.001) patients. Interestingly, the significantly higher levels of antibodies among *P. vivax* infected Thai compared to Chinese patients (P = 0.02) were observed. 

#### Anti-P. vivax AMA-1

The mean level of antibodies against PvAMA-1 among healthy controls living outside the *P. vivax* endemic area was very low both in Thai (OD = 0.1) and in Chinese populations (OD = 0.03) (Figure 1C(Fig. 1)). However, the level was significantly higher among the Thai population compared to the Chinese population (P < 0.000). There was a higher level among Thai (0.2) than Chinese immune controls (0.1) (P < 0.001). There was no significant difference in the level of antibodies against PvAMA-1 in the healthy Thai donors living inside and outside endemic areas (P > 0.05) in contrast to that found in the Chinese donors (P < 0.001). In acute *P. vivax* infection, the level was significantly higher compared to naïve controls (Thai P =< 0.001, Chinese P < 0.0.001). However, among *P. vivax* patients, we did not find significant difference between Thai (0.3) and Chinese (0.2) populations (P > 0.05). We also found a sample of *P. vivax* infected patient that had obviously high level of antibodies to PvAMA-1, so this was excluded from the baseline value.

#### Cross reactivity between P. vivax and P. falciparum antigens

To examine the immune cross-reactivity between *P. vivax* and *P. falciparum* antigens, the *P. vivax* and *P. falciparum* extracted antigens were used as coated antigens on the ELISA plates. The median percentage of baseline level antibodies in naïve controls among Chinese was very low (OD = 0.003) and was significantly lower than that of Thai (OD = 0.04) donors (MD = 0.07, 95 % CI = 0.04-0.1, P < 0.001) (Figure 1D(Fig. 1)). Moreover, significantly higher levels of antibodies against *P. falciparum* antigens were observed in immune controls between Thai (0.18) and Chinese (0.01) (MD = 0.6, 95 %CI = 0.45-0.75). After infection with *P. vivax*, patients had higher levels of antibodies against *P. falciparum* in Thai patients (OD = 0.36) than in Chinese patients (OD = 0.07).

## Discussion

In this study, we provide evidence that Thai villagers having had *P. vivax* infection and living in the endemic area did not produce high level of anti-*P. vivax*-specific antibodies (Jangpatarapongsa et al., 2006[7]) as tested with total proteins extracted from the parasites. We confirmed the presence of antibodies to *P. vivax* antigens by testing with recombinant *P. vivax* proteins, i.e. PvMSP1(19) and PvAMA1, then compared the level of natural antibodies between two *P. vivax* endemic areas in China and in Thailand. The species specific antibodies could tell us the cross-reactivity between* P. falciparum* and *P. vivax* which led to the examination of epidemiological status in those areas. 

Previously, a passive transfer of immune IgG to Gambian children was shown to provide protection (McGregor, 1964[12]). The immunity against blood stage of *P. falciparum* infection is associated with class and subclass of IgG antibody (Shi et al., 1999[21]). Similarly, IgG1 and IgG3 are predominant among *P. vivax*-infected patients with history of malaria (Pinto et al., 2001[16]). Recent study has shown anti-*P. vivax* merozoite surface protein 1 (MSP-1) IgG among subjects with distinct degrees of malaria exposure in endemic area. The IgG1 and IgG3 against *P. vivax* are higher among the subjects with one year-exposure period than the group with longer years of exposure (Morais et al., 2005[14]). PvMSP-1(19) is shown to induce immunity in non-human primates (Rosa et al., 2006[19]). 

Our study showed no significant difference of base line level anti-*P. vivax* antibodies among Chinese and Thai patients. We found higher levels of anti-*P. vivax* antibodies in Chinese than in Thai immune controls. Moreover, this level was very high in similarity to that shown in the acute *P. vivax* infection. This could be that *P. vivax* endemicity in China was greater than in Thailand, although the two areas in both countries are hypo-endemic. 

However, in contrast to what we found in anti-*P. vivax* antibodies, the base line level of anti-MSP-1 and anti-AMA-1 antibodies was significantly lower in naïve Chinese controls than in naïve Thai controls. A possible reason could be that some of these malaria naïve Thai donors might have had malaria experiences but were asymptomatic and, therefore, there were lower levels of antibodies that persisted in their circulation. 

In our study, we found the level of anti-MSP-1 antibodies was significantly lower in immune controls and acute *P. vivax* infected Chinese. A possible reason is that the recombinant protein rMSP-1(19) was obtained from the *P. vivax* parasites infecting Thai patients. There may be difference in the gene sequence of *P. vivax* MSP-1(19) between those from Thailand and those from China (Birkenmeyer et al., 2010[1]).

Crude *P. falciparum* antigen was used in this study to determine the species specific antibody and the cross-reactivity. We found very low levels of base line anti-*P. falciparum* antibodies among naïve controls and immune controls of Chinese donors. After infection with *P. vivax*, Chinese patients had somewhat higher levels of anti-*P. falciparum* antibodies. These results were in contrast to those found in Thailand. Higher levels of base line anti-*P. falciparum* antibody was found among naïve and immune Thai controls. Moreover, development of anti-*P. falciparum* antibodies in *P. vivax* infected patients in Thailand were much higher than that in China. Taken together, this suggests that a cross-reactivity among epitopes/antigens between the two malaria species do exist. However, it does not over rule the fact that the anti-*P.falciparum* antibody may be maintained in the *P. vivax*-infected Thai patients since both parasites are common in the area. Our findings led to the hypothesis that a protection against severe malaria caused by *P. falciparum* might be obtained via vaccination with a common antigen(s) of a benign malaria parasite such as *P. vivax*.

## Acknowledgements

We thank Dr. Hua Hai Yong for supporting healthy donors plasma, Drs. Chen Chong Xin, Dr. Zhu Mu Shan, Dr. Gao Lai, Dr. Luo Wen Wen for immune controls and malaria patients’ sera. 

This work was supported by the Office of Higher Education Commission and Mahidol University under the National Research Universities. The Thailand Research Fund (BRG498009) to RU, and D43TW006571 to LC from The Fogarty International Center, NIH, USA.

## Conflict of interest

The authors declare that they have no conflict of interest.

## Notes

Dr. Rachanee Udomsangpetch (E-mail: rachanee.udo@mahidol.ac.th) and Dr. Baiqing Li (E-mail: bb_bqli@yahoo.com) contributed equally as corresponding authors.

Hui Xia and Qiang Fang contributed equally to this work.

## Figures and Tables

**Table 1:**
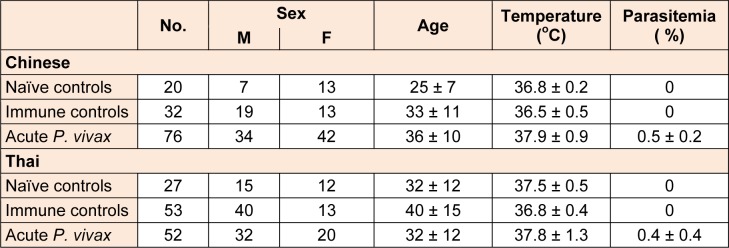
Information and clinical data of *P. vivax* patients, immune and naïve controls

**Table 2:**
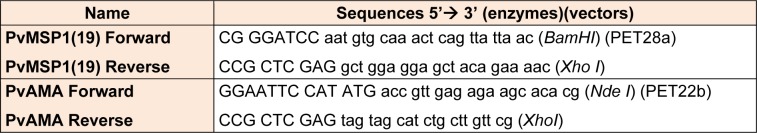
PCR primers and sequences
